# KRAS, EGFR, PDGFR-α, KIT and COX-2 status in carcinoma showing thymus-like elements (CASTLE)

**DOI:** 10.1186/1746-1596-9-116

**Published:** 2014-06-16

**Authors:** Lothar Veits, Rupert Schupfner, Petra Hufnagel, Roland Penzel, Jens Freitag, Philipp Ströbel, Michael A Kern, Sören Schröder, Nikolaus Neuhold, Kurt W Schmid, Peter Schirmacher, Arndt Hartmann, Ralf J Rieker

**Affiliations:** 1 Institute of Pathology, Klinikum Bayreuth, Preuschwitzerstraße 101, Bayreuth 95445, Germany; 2 Clinic of Trauma and Reconstructive Surgery, Klinikum Bayreuth, Preuschwitzerstraße 101, Bayreuth 95445, Germany; 3 Pathologielabor Zams, Klostergasse 1, Zams 6511, Austria; 4 Department of Pathology, University Hospital, INF 220/221, Heidelberg 69120, Germany; 5 General practitioner, Moselstraße 1, Oberbettringen 73529, Germany; 6 Department of Pathology, University Hospital, Robert-Koch-Straße 40, Göttingen 37075, Germany; 7 Institute of Pathology/Lademannbogen Laboratories, Lademannbogen 61-63, Hamburg 22339, Germany; 8 Pathologisch-Bakteriologisches Institut, Kaiserin-Elisabeth-Spital, Huglgasse 1-3, Vienna 1150, Austria; 9 Department of Pathology and Neuropathology, University Hospital Essen, Hufelandstraße 55, Essen 45122, Germany; 10 Department of Pathology, University Hospital Erlangen, Krankenhausstr. 10-12, Erlangen 91054, Germany

**Keywords:** CASTLE, Thymic carcinoma, Mutational analysis, Immunohistochemistry, Thyroid gland

## Abstract

**Background:**

CASTLE (Carcinoma showing thymus-like elements) is a rare malignant neoplasm of the thyroid resembling lymphoepithelioma-like and squamous cell carcinoma of the thymus with different biological behaviour and a better prognosis than anaplastic carcinoma of the thyroid.

**Methods:**

We retrospectively investigated 6 cases of this very rare neoplasm in order to investigate the mutational status of *KRAS*, *EGFR*, *PDGFR-α* and *KIT*, as well as the immunohistochemical expression pattern of CD117, EGFR and COX-2, and possibly find new therapeutic targets.

**Results:**

Diagnosis was confirmed by a moderate to strong expression of CD5, CD117 and CK5/6, whereas thyroglobulin, calcitonin and TTF-1 were negative in all cases. Tumors were also positive for COX-2 and in nearly all cases for EGFR. In four cases single nucleotide polymorphisms (SNPs) could be detected in exon 12 of the *PDGFR-α* gene (rs1873778), in three cases SNPs were found in exon 20 of the *EGFR* gene (rs1050171). No mutations were found in the *KIT* and *KRAS* gene.

**Conclusions:**

All tumors showed a COX-2 expression as well as an EGFR expression except for one case and a wild-type *KRAS* status. No activating mutations in the *EGFR, KIT* and *PDGFR-α* gene could be detected. Our data may indicate a potential for targeted therapies, but if these therapeutic strategies are of benefit in CASTLE remains to be determined.

**Virtual Slides:**

The virtual slide(s) for this article can be found here: http://www.diagnosticpathology.diagnomx.eu/vs/1658499296115016

## Background

Carcinoma showing thymus-like elements (CASTLE) is a rare malignant tumor of the thyroid gland. It was first described by Miyauchi et al. in 1985 as “intrathyreoidal epithelial thymoma” [1], is thought to arise from ectopic thymic tissue within the thyroid gland or rudimentary branchial pouches along the thymic line and was named CASTLE by Chan and Rosai in 1991 [2]. CASTLE-tumors belong to a group of ectopic tumors of thymic differentiation: Ectopic hamartomatous thymoma, ectopic cervical thymoma, spindle epithelial tumors with thymus like differentiation (SETTLE) and carcinoma showing thymic-like elements (CASTLE). Although CASTLE shows some tendencies for squamous differentiation, it has newly been adopted as an independent entity by the recent WHO classification [3]. In contrast to ectopic thymomas, SETTLE and CASTLE are always malignant and therefore the exact diagnosis is crucial in consideration of prognosis and treatment. CASTLE tumors have characteristic clinical and histological features. They are tumor of the adults in the fifth decade of life and often invade the adjacent soft tissue. Metastases into the regional lymph nodes are common. The largest study yet performed comprised 25 cases [4]. According to this study it is likely that some thyroid cancers, especially those with squamous differentiation may have been treated incorrectly based on an improper classification. For our study we collected 6 cases of this rare malignancy and evaluated possible therapeutic options.

## Methods

In our study we included 6 patients suffering from CASTLE, 3 women and 3 men, diagnosed between 1997 and 2007. The median age was 60 years ranging from 30 to 68 years.

### Tissue microarrays and immunohistochemistry

Tissue microarrays (TMA) were performed from the paraffin-wax-embedded blocks of the tumor specimens. A tissue arrayer device (Beecher Instruments, Sun Prairie, WI, USA) was used. All the investigated cases were reviewed and representative tumor areas were marked in the corresponding paraffin wax blocks, of which at least two were sampled. The diameter of the cylinders was 1.2 mm. For immunohistochemistry 5 μm sections on poly-L-lysine-coated slides were used after drying in an oven, followed by dewaxing and peroxidase blocking with 1% H_2_O_2_. The specificity of immunostaining was tested by replacing primary antisera with normal rabbit serum. No additional pre-treatment procedure for antigen retrieval was done. The immunoreactions were performed with the following primary monoclonal antibodies: Anti-COX-2 (monoclonal rabbit IgG; Santa Cruz, USA; 1:50), anti-CD5 (monoclonal mouse IgG; Novocastra, Leica Microsystems, Wetzlar, Germany; 1:25), anti-TTF-1 (monoclonal mouse, IgG, SPT24; Menarini, Florence, Italy; 1:100), anti-CK 5/6 (monoclonal mouse IgG, M7237; Dako Cytomation, Glostrup, Denmark; 1:50), anti-Ki67 (monoclonal mouse IgG, K-2, Zytomed, Berlin, Germany; ready-to-use). Primary polyclonal antibodies used were: CD117 (polyclonal rabbit, A4502; Dako Cytomation, Glostrup, Denmark; 1:50), anti-thyroglobulin (polyclonal rabbit, A0251; Dako Cytomation, Glostrup, Denmark; 1:80000) and anti-calcitonin (polyclonal rabbit, A0576; Dako Cytomation, Glostrup, Denmark; 1:4000). After washing steps signal detection was performed (EnVision + system-HRP, AEC, Dako Hamburg, Germany; 1:200) followed by counterstaining in hemalaun. Detection of EGFR was performed with the EGFR pharmDX kit (Dako Hamburg, Germany) according to manufacturer’s instructions.

The presence of clearly visible dark brown precipitation was considered as immunoreaction, evaluated by a score for staining intensity (0: no staining; 1: weak; 2: moderate; 3: strong reaction intensity).

### Mutational analysis of *KIT, PDGR-α, EGFR, KRAS*

Cores of tumor tissue selected from regions on correspondent Hematoxylin-Eosin slides were manually punched out from the donor blocks. DNA was extracted from paraffin-embedded tissue sections using EZ1 DNA tissue Kit (Qiagen, Hilden Germany) after pre-treatment with proteinase K (Qiagen, Hilden Germany). 150 ng DNA were used for PCR amplification and all PCR reactions were run at a final volume of 25 μl (Red Jump MasterMix, Sigma-Aldrich). Exons 9, 11, 13 and 17 of *KIT*, exons 10, 12, 14 und 18 of *PDGFR-α*, exons 18, 19, 20 and 21 of *EGFR* and exons 2 and 3 of *KRAS* were evaluated for the presence of mutations by PCR amplification and direct sequencing. The primer pairs used for PCR amplification and direct sequencing are shown in Table 1. After estimation of DNA concentration by spectroscopy (GeneQuant) relevant exons were amplified by PCR under standard conditions with an annealing temperature of 50°C for Exon 17 (*KIT*), 55°C for Exon 2 and 3 (*KRAS*), Exon 9 (*KIT*) and Exon 18 (*PDGFR*), 60°C for Exon 18, 19, 20 and 21 (*EGFR*) and Exon 11 and 13 (*KIT*) as well as Exon 10, 12 and 14 (*PDGFR*) with the corresponding primers. The PCR products were then separated on agarose gel containing ethidiumbromide and visualized under ultraviolet light. Afterward PCR products were purified using high pure PCR product purification kit (Roche Diagnostics, Penzberg, Germany) and then sent to Qiagen (Hilden, Germany) for sequencing according to Sanger method.

**Table 1:** Primer sequences for KIT, PDGR, EGFR, KRAS PCR amplification

**KIT9F 5′GCC ACA TCC CAA GTG TTT TAT G**	**KIT9R 5′GAG CCT AAA CAT CCC CTT AAA TTG**
**KIT11F** 5′CCA GAG TGC TCT AAT GAC TG	**KIT11R** 5′AGC CCC TGT TTC ATA CTG AC
**KIT13F** 5′CTT GAC ATC AGT TTG CCA GTT GT	**KIT13R** 5′GAC AGA CAA TAA AAG GCA GCT TG
**KIT14F** 5′CTC ACC TTC TTT CTA ACC TTT TCT T	**KIT14R** 5′CCC ATG AAC TGC CTG TCA AC
**KIT17F** 5′ATG GTT TTC TTT TCT CCT CC	**KIT17R** 5′TAC ATT ATG AAA GTC ACA GG
**PDGFRA10F** 5′GGC CCT ATA CTT AGG CCC TTT T	**PDGFRA10R** 5′TGT CCT GAC TGT TGA GGA ACT
**PDGFRA12F** 5′CTC TGG TGC ACT GGG ACT TT	**PDGFRA12R** 5′GCA AGG GAA AAG GGA GTC TT
**PDGFRA14F** 5′TCT GAG AAC AGG AAG TTG GTA GC	**PDGFRA14R** 5′CCA GTG AAA ATC CTC ACT CCA
**PDGFRA18F** 5′TCT TGC AGG GGT GAT GCT AT	**PDGFRA18R** 5′AGA AGC AAC ACC TGA CTT TAG AGA TTA
**EGFR18F** 5′GCT GAG GTG ACC CTT GTC TC	**EGFR18R** 5′ACA GCT TGC AAG GAC TCT GG
**EGFR19F** 5′GCT GGT AAC ATC CAC CCA GA	**EGFR19R** 5′GAG AAA AGG TGG GCC TGA G
**EGFR 20 F** 5′CAT GTG CCC CTG CTT CTG	**EGFR 20R** 5′GAT CCT GGT TCC TTA TCT CC
**EGFR21F** 5′CCT CAC AGC AGG GTC TTC TC	**EGFR21R** 5′CCT GGT GTC AGG AAA ATG CT
**KRAS2F** 5′GTG TGA CAT GTT CTA ATA TAG TCA	**KRAS2R** 5′GAA TGG TCC TGC ACC AGT AA
**KRAS3F** 5′CCA GAC TGT GTT TCT CCC TTC	**KRAS3R** 5′TGC ATG GCA TTA GCA AAG AC

The different exons of *KIT, PDGFR, EGFR* and *KRAS* are written in bold letters.

### Ethical approval

This study was approved by the ethics committee of the University Hospital Heidelberg (reference number: 206/2005).

## Results

### Clinical and histological characteristics

The primary symptom was a palpable node in 2 patients and an adenomatous goitre as well as a paresis of the recurrent nerve in 1 case, respectively. In 3 cases no case history was available. 5 tumours were located in the thyroid and 1 was partially extrathyroidal, the latter with the majority of the tumor mass in the thyroid gland.

**Figure 1:**
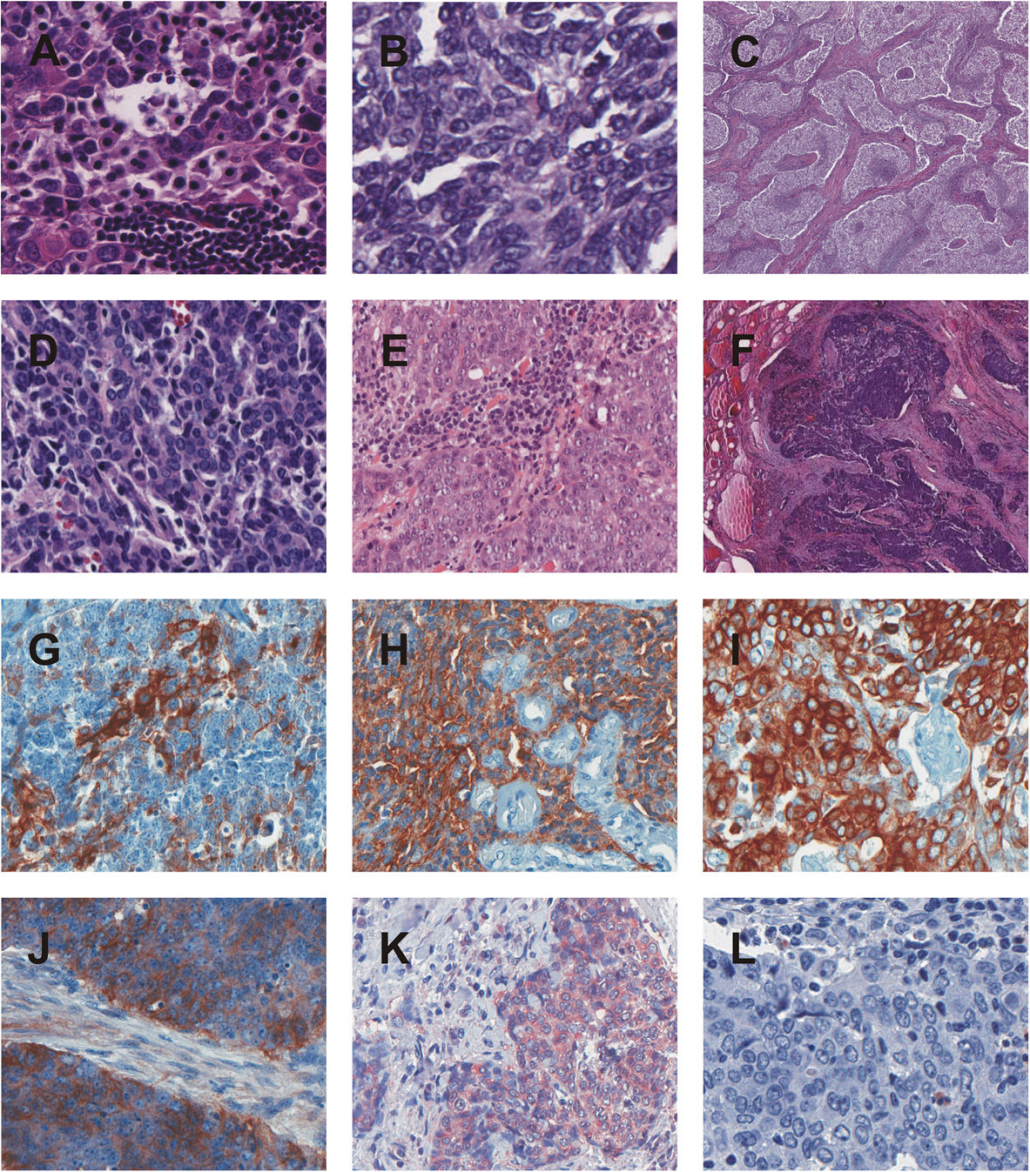
**HE and immunohistochemical stainings. A**-**F**. HE staining case 1–6. **A**. HE case 1, **B**. HE case 2, **C**. HE case 3, **D**. HE case 4, **E**. HE case 5, **F**. HE case 6. **G**-**L**. Immunohistochemical staining case 1–6 for CD5, CD117, CK5/6, COX-2, EGFR and TTF-1. **G**. CD5 case 1. **H**. CD117 case 5. **I**. CK5/6 case 4. **J**. EGFR case 6. **K**. COX-2 case 2. **L**. TTF-1 case 3.

Local tumor stage was T2 in one case, T3 in 3 cases, the remaining cases T4. In 3 cases no lymph nodes were affected, 3 patients already developed lymph node metastases. All cases underwent curative hemi- or total thyroidectomy. 5 patients received postoperative radiotherapy, 1 received a combined radio- and chemotherapy. The median time of follow-up observation was 18.5 months, with a range from 2 to 36 months. During this time, 1 patient developed local recurrence 2 months after primary operation and underwent a second tumorectomy with bilateral neck dissection and transsternal lymphadenectomy. The remaining patients were recurrence-free (see Table 2). By histology the resected specimen showed fibrous septa with lobulation and surrounding tumor islands of various size (see Figure 1C + F). Especially the peripheral tumor tissue was infiltrated by small lymphocytes (see Figure 1A,B,D,E). Scattered blood vessels were found, surrounded by fibrous connective tissue.Tumor cells were varying in shape: Irregular cells as well as spindle-shaped and epithelioid cells were observed. Occasionally, squamoid or spindle-shaped epithelial cells with whorls resembling Hassall’s corpuscles were noticed. The cytoplasm of the tumor cells was pale and nuclei were clearly evident and slightly oval (see Figure 1A - F).

**Table 2:** Patient characteristics and tumor progression

**No.**	**Stage**	**Therapy**	**Progression**
1	pT4, N1b (18/21), MX, R0	Total thyroidectomy, left central neck dissection, resection of the left recurrent laryngeal nerve and the left internal jugular vein, chemotherapy, radiation (50 Gy) After recurrence: Tumorectomy, bilateral neck dissection, transsternal lymphadenectomy	Local recurrence after 2 months
2	pT3, N0 (0/5), MX, R0	Thyroidectomy, cervico-central lymphadenectomy	No recurrence (time of observation: 36 months)
3	pT3, N0, MX	Subtotal thyroidectomy, local telecobalttherapy of the neck (60 Gy)	No recurrence (time of observation: 19 months)
4	pT2, N0 (0/7), MX	Hemithyroidectomy right, then restthyroidectomy, microdissection of the cervicocentral compartment, radiation of the thyroidbed (59,4 Gy)	No recurrence (time of observation: 15 months)
5	pT3, N1 (1/19), MX	En bloc resection with the right lobe, neurolysis N.laryngeus recurrens right, neck dissection right, supraclavicular cervical lateral dissection, radiation (50 Gy)	No recurrence (time of observation: 17 months)
6	pT4a, N1 (1/5), M0, R0	Total thyroidectomy, neck dissection, radiation of the neck region (50 Gy)	No recurrence (time of observation: 22 months)

*No.* case number; *Stage* TNM classification.

### Immunohistochemistry

All CASTLE cases expressed CD5 in a moderate to strong reaction and CD117 in predominantly weak to moderate intensity in the epithelial component. Only in one case a strong staining signal for CD117 could be detected. (see Figure 1G + H). Staining with CK5/6 showed weak to strong reactions in all samples (see Figure 1I). EGFR showed a strong reactivity in three, weak staining intensity in two samples and no staining pattern in one case (see Figure 1J). COX-2 was detected positively in all cases with mainly moderate expression patterns (see Figure 1 K). The expression of Ki67 showed varying results between 2% and 85%. The staining results for CD117, EGFR, COX-2 and Ki67 are shown summarized in Table 3. All cases were negative for calcitonin, thyroglobulin and TTF-1 (Figure 1L).

**Table 3:** Immunohistochemical expression pattern

**No.**	**CD117**	**EGFR**	**COX-2**	**Ki67 (%)**
1	1	1	2	17
2	2	3	2	7
3	1	0	3	2
4	1	3	1	12
5	3	1	2	53
6	2	3	2	85

*No*. case number; *0* no staining, *1* weak, *2* moderate, *3* strong reaction intensity.

### Mutational analysis of KIT, PDGFR-α, EGFR, KRAS

Mutational analysis of *KIT, PDGFR-α, EGFR* and *KRAS* was possible in only 4 cases due to inapplicable DNA in case number 2 and 3.

**Figure 2:**
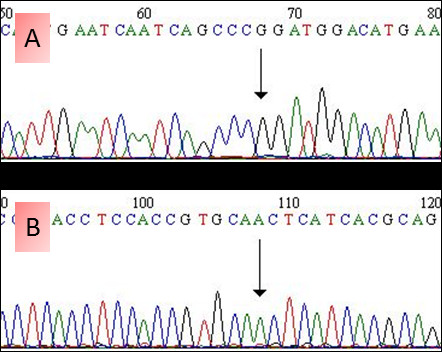
**SNPs in *PDGFR *and *EGFR *genes. A**. Forward chromatogram showing c.A1701G, predicting p.P567P in the *PDGFR* gene (db SNP = rs1873778). **B**. Forward chromatogram showing c.G2361A, predicting p.Q787Q in the *EGFR* gene (db SNP = rs1050171).

Mutation analysis of all four investigated cases revealed the absence of an activating mutation in the *PDGFR-α* gene of exon 10, 12, 14 and 18. Nevertheless, in all four cases a silent single nucleotide polymorphism with a base substitution (CCA > CCG) in exon 12 at codon 567 (p.P567P) of the *PDGFR-α* gene was observed. This change has already been described in the SNP database with the number rs1873778 (Figure 2A). In 3 (3/4) patients a silent single nucleotide polymorphism with a base substitution (CAG > CAA) in the *EGFR* gene of exon 20 at position 787 (p.Q787Q) was observed, which has also already been described in the SNP database with the number rs1050171 (Figure 2B). No mutation associated with drug sensitivity in the hotspot exons 18, 19, 20 and 21 was detected. No patient had detectable sequence variants for *KIT* in the hotspot region of exon 9, 11, 13 and 17. Also no sequence variants were found in the sequenced exons of the *KRAS* gene (Table 4).

**Table 4:** Sequence variants in the PDGFR-α, EGFR, KRAS and KIT genes

**Gene**	**Exon**	**Nucleotide alt.**	**Amino acid subst.**	**No.**	**Interpretation**	**dbSNP**
*PDGFR-α*	10	-	-	4	wild type	-
	12	c.A1701G	p.P567P	4	SNP	rs1873778
	14	-	-	4	wild type	-
	18	-	-	4	wild type	-
*EGFR*	18	-	-	4	wild type	-
	19	-	-	4	wild type	-
	20	c.G2361A	p.Q787Q	3	SNP	rs1050171
	20	-	-	1	wild type	-
	21	-	-	4	wild type	-
*KRAS*	2	-	-	4	wild type	-
	3	-	-	4	wild type	-
*KIT*	9	-	-	4	wild type	-
	11	-	-	4	wild type	-
	13	-	-	4	wild type	-
	17	-	-	4	wild type	-

*No*. Number of patients; *dbSNP*http://www.ncbi.nlm.nih.gov/SNP/; *SNP* single nucleotide polymorphism; *Nucleotide alt*. Nucleotide alteration; *Amino acid subst*. Amino acid substitution.

## Discussion

CASTLE tumors are rare malignant tumors occurring in the thyroid, but the prognosis and the concomitant long term survival rate is better than for other aggressive advanced thyroid neoplasms [1,2,5]. Among other things and according to the so far largest study of CASTLE-tumors comprising 25 cases [4,6] a significant number may have been incorrectly classified, especially as squamous cell carcinoma of the thyroid. In this study Ito et al. showed a survival rate of 90% after 5 years and 82% after 10 years. Patients without lymph node metastasis had an overall survival rate of 100% after 5 years, those with lymph node metastasis 76% after 5 and 57% after 10 years. One of our patients with initial lymph node metastasis had recurrence after 2 months. No recurrence during an observation period of up to 73 months occurred in the other patients. Interestingly, the expression of Ki67 showed varying results between 2% and 85%, but could not be linked to the presence of lymph node metastases or advanced tumor stages, as the only case in which a tumor recurrence was observed showed a low proliferation rate (compared to e.g. striking 85% in case number 6 with no recurrence during an observation period of 22 months).

Because a diagnosis of CASTLE is difficult before resection of the tumor, patients are usually treated according to the standards of thyroid gland carcinoma with surgical removal of all or part of the thyroid gland with or without neck dissection. Depending on the tumor entity and stage postoperative therapy is often indicated. 5 of our patients underwent radiotherapy, 1 combined radiochemotherapy.

We have recently described chromosomal imbalances in CASTLE similar to those found in thymomas and thymic carcinomas [7]. Given the morphological, immunohistochemical and genetic similarities with thymic carcinomas, it seems reasonable to assume that treatment of advanced CASTLE should follow the guidelines for thymic tumors and patients may benefit also from evolving therapeutic options for patients with thymic tumors [8-13].

CD117 is expressed in about 86% of thymic carcinomas [14], but *KIT* gene mutations are uncommon. So far, only few cases of metastasizing thymic carcinoma with an activating *KIT* mutation and a partial response to treatment with Imatinib have been described [15,16]. In our analysis, all CASTLE tumors showed positive staining results for CD117, but no mutations of the *KIT* gene.

Membrane associated EGFR expression has been seen in thymomas as well as in thymic carcinomas. Girard summarized eight studies [17], which investigated EGFR expression levels in thymic malignancies [18-25] using immunohistochemistry. In this meta-analysis, EGFR was overexpressed in 70% of thymomas and 53% of thymic carcinomas, but there was no correlation between EGFR expression and thymic tumor type. Activating *EGFR* mutations seem to be exceptionally rare in thymic carcinomas with only few reported *EGFR* missense mutations in exon 21 (p.L858R in two cases and p.G863D in one case) [23,26]. In the present series, five tumors showed weak to strong immunohistochemical staining results for EGFR. Two of the four cases, in which DNA-extraction was successful, showed a weak positivity for EGFR, the other two cases showed a strong signal. In three cases mutational analysis revealed a single nucleotide polymorphism (SNP) in the *EGFR* gene of Exon 20 (p.Q787Q), but no activating *EGFR* gene mutation could be found.

It has been shown that a therapeutic effect can be seen after the use of EGFR-inhibitors in non-small cell lung cancers, even if there is no activating mutation in the *EGFR* gene [27]. In a small series of advanced thymic tumours only a slight therapeutic effect could be demonstrated with Gefitinib [28].

Randomised clinical trials have demonstrated the potential of COX-inhibitors in tumor prevention [29]. Malka et al. showed that inhibition of COX-2 could play a possible therapeutic role in other tumor entities, for instance hepatocellular carcinoma [30]. In particular, the combination of COX-2 inhibitors and EGFR-inhibitors seems to increase the therapeutic effect in colon or cervical carcinoma [31-34]. COX-2 is frequently upregulated in thymomas and thymic carcinomas [11,12]. In our study all tumors expressed COX-2 raising the possibility to use COX-2 inhibitors in advanced cases.

Although in our investigation all four cases showed SNPs in the *PDGFR-α* gene of exon 12, no activating mutations in the *PDGFR-α* gene and no mutations in Codon 12 and 13 of the *KRAS* gene were detected.

## Conclusion

As already mentioned above, CASTLE shows some similarity to thymic carcinoma. However, in the latter targeted therapy is difficult and somehow limited in advanced tumors as there are only few reported cases in which targeted therapy was of some therapeutic benefit. Ströbel et al., for example, suggest that in cases with advanced thymic carcinoma treatment with the multi-kinase inhibitor Sunitinib, that targets VEGFR1–3, PDGFR-*α*, c-KIT, FLT3, colony stimulating factor-1 (CSF1) and the RET receptor, is more effective than therapeutic regimes using single-target molecular therapies like Gefitinib, even though there are no activating mutations in the *EGFR, KIT* and *PDGFR-α* gene [35]. In conclusion, our data may indicate a potential for targeted therapies, but if these therapeutic strategies are of benefit in CASTLE remains to be determined.

## Competing interests

The authors declare that they have no competing interests.

## Authors’ contributions

LV generated the study concept and design, evaluated the HE- and immunohistochemical stains, analysed and interpreted the data, confirmed the diagnosis and drafted the manuscript. RS critically revised the manuscript for important intellectual content and participated in the design of the study. PH carried out the molecular genetic studies, participated in the sequence alignment and analysed and interpreted the data. RP participated in the molecular genetic studies and critically revised the manuscript for important intellectual content. JF critically revised the manuscript for important intellectual content and participated in the analysis of the data. PS critically revised the manuscript for important intellectual content and participated in the study design and concept. MK carried out the immunoassays. SS critically revised the manuscript for important intellectual content and participated in the study design and concept. NN critically revised the manuscript for important intellectual content and participated in the study design and concept. KS participated in the study design and concept and critically revised the manuscript for important intellectual content. PS critically revised the manuscript for important intellectual content. AH critically revised the manuscript for important intellectual content. RR critically revised the manuscript for important intellectual content, participated in the design of the study and evaluated the HE- and immunohistochemical stains, confirmed the diagnosis. All authors read and approved the final manuscript.

## Authors’ information

Lothar Veits and Rupert Schupfner share first authorship.
